# A new class of tunable hypersonic phononic crystals based on polymer-tethered colloids

**DOI:** 10.1038/ncomms9309

**Published:** 2015-09-22

**Authors:** E. Alonso-Redondo, M. Schmitt, Z. Urbach, C. M. Hui, R. Sainidou, P. Rembert, K. Matyjaszewski, M. R. Bockstaller, G. Fytas

**Affiliations:** 1 Max Planck Institute for Polymer Research, Ackermannweg 10, 55128 Mainz, Germany; 2 Department of Materials Science and Engineering, Carnegie Mellon University, 5000 Forbes Avenue, Pittsburgh, Pennsylvania 15213, USA; 3 Department of Chemistry, Carnegie Mellon University, 4400 Fifth Avenue, Pittsburgh, Pennsylvania 15213, USA; 4 Laboratoire Ondes et Milieux Complexes, UMR CNRS 6294, University of Le Havre, 75 Rue Bellot, 76600 Le Havre, France; 5 Department of Materials Science, FORTH-IESL, PO Box 1527, 71110 Heraklion, Greece

## Abstract

The design and engineering of hybrid materials exhibiting tailored phononic band gaps are fundamentally relevant to innovative material technologies in areas ranging from acoustics to thermo-optic devices. Phononic hybridization gaps, originating from the anti-crossing between local resonant and propagating modes, have attracted particular interest because of their relative robustness to structural disorder and the associated benefit to ‘manufacturability'. Although hybridization gap materials are well known, their economic fabrication and efficient control of the gap frequency have remained elusive because of the limited property variability and expensive fabrication methodologies. Here we report a new strategy to realize hybridization gap materials by harnessing the ‘anisotropic elasticity' across the particle–polymer interface in densely polymer-tethered colloidal particles. Theoretical and Brillouin scattering analysis confirm both the robustness to disorder and the tunability of the resulting hybridization gap and provide guidelines for the economic synthesis of new materials with deliberately controlled gap position and width frequencies.

## Abstract (web summary)

Hybridization-type band gaps are known to persist in phononic crystals, but their fabrication remains challenging for all-solid hypersonic composites. Here, the authors utilize the elastic anisotropy at the interface of polymer-tethered colloidal particles to control phonon propagation in GHz regime.

## Graphical abstract

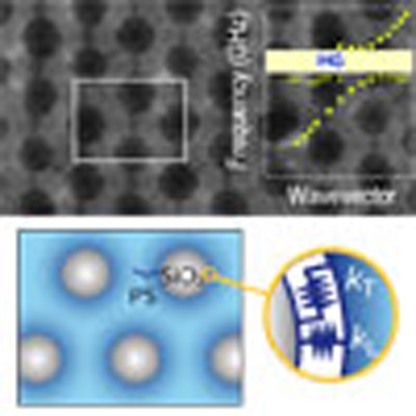

Phononic crystals (PnC), that is, composite materials in which a periodic distribution of elastic parameters facilitates control of the propagation of phonons, hold the promise to enable transformative material technologies in areas ranging from acoustic and thermal cloaking to thermoelectric devices^1^,^2^,^3^,^4^,^5^. Realizing these opportunities requires strategies to deliberately ‘engineer' the phononic band structure of materials in the frequency range of interest. The typical approach involves the exploitation of Bragg-type phononic band gaps (BGs) that result from the destructive interference of waves in periodic media^6^ (Fig. 1a). Because the central frequency of a BG is directly related to the periodicity of the structure, that is, *f*_BG_∼*c*/2*a* (where *a* and *c* denote the lattice parameter and the propagation velocity of elastic waves in the composite, respectively), the control of high-frequency phonons implies the ability to tailor the microstructure of hybrid materials on nanometer scales. Self-assembly processes have attracted particular attention as viable fabrication methodology to realize hybrid materials with appropriate periodicity in the nanoscale. For example, the assembly of colloidal particles into fcc-type crystal structures has been shown to enable the fabrication of hypersonic PnC materials to control phonons in the GHz regime^7^,^8^,^9^. However, the sensitivity of BG formation to structural disorder has limited the application of self-assembly methods that are generally susceptible to defect formation. Opportunities to overcome this challenge are provided by the formation of the so-called hybridization-type gaps (HGs), resulting from the avoided crossing of two bands of the same symmetry^10^,^11^,^12^,^13^,^14^. Figure 1 contrasts the characteristics of BG and HG formation.

Often, at least one of these crossing bands originates from localized resonance modes of the individual particles (Fig. 1b). In this case, the frequency of the resulting HG is determined by the particle's geometric and elastic characteristics, that is, *f*_HG_∼*c*_p_/2*L* (where *L* denotes a characteristic length of the particle and *c*_p_ is the propagation velocity characterizing the resonance mode). As a consequence of the dependence on local resonances, HGs are robust to disorder^10^,^14^, and can be tuned over a wider range of frequencies. Because *f*_HG_ is related to the length scale of individual particles rather than lattice periods, the anti-crossing mechanism is able to produce phononic band gaps at lower frequencies as compared with BG-analogues—thus providing opportunities to further expand the range of frequencies over which control of phonon propagation can be accomplished. HG formation has been experimentally demonstrated in a range of ‘particulate systems' such as polymer spheres in water-like hosts^10^,^13^,^14^ or ‘metal-in-polymer' systems, such as millimetre-scale layered spheres. The latter were first applied by Sheng and co-workers to realize sonic structures operating in the kHz range^15^. However, although these structures did prove the concept of HG formation, they are of limited use for PnC applications because of their lack of mechanical stability (liquid suspensions), pronounced optical absorption (in the case of metals) that hampers any type of application based on elasto-optical coupling, as well as fabrication cost and limitation to the kHz frequency regime (micro-fabricated structures). Therefore, a viable methodology for the fabrication of HG materials, which lends itself to the scalable production of robust all-solid PnCs active over a wide high-frequency range, remains an outstanding challenge.

Here we demonstrate a new approach to facilitate HG formation in polymer nanocomposite materials that holds the promise to provide transformative opportunities for the use of self-assembly methods for the scalable fabrication of phononic materials, overcoming the limitations of existing methods to HG formation. This approach harnesses local anisotropy of the elastic parameters across the particle/polymer interface in colloidal hybrid particles rather than the density mismatch between constituents (appearing in metal-in-polymer all-solid composites). In particular, nanocomposite films prepared by simple solution casting of densely polymer-tethered colloids are shown to exhibit a robust HG in the hypersonic range that is retained in the absence of long-range order. Rigorous theoretical calculations based on multiple-scattering theory (using imperfect boundary conditions, IBCs) reveal the mechanism of HG formation in particulate systems with anisotropic elastic coupling and provide guidelines for the synthesis of polymer-tethered colloids capable of forming HG at deliberately chosen frequencies in the hypersonic range.

## Results

### Polymer-tethered colloids and their assemblies

The silica (SiO_2_)-polystyrene (PS) brush particles, with average radius *R*_c_=57±6 nm of the SiO_2_ core determined by transmission electron microscopy (TEM), were synthesized using surface-initiated atom transfer radical polymerization as described previously^16^,^17^. The polymer grafting density *σ*=0.5–0.6 chains per nm^2^ was determined by elemental analysis and thermogravimetry. The graft characteristics of the particle systems, grafting density *σ* and degree of polymerization *N* (130–980) of the surface tethered chains are listed in Table 1 (sample ID: DP*N*). The centre-to-centre distance, *d*=2(*R*_c_+*h*), where *h* is the brush height (Fig. 2a), is also listed in Table 1.

Particle brush films with a thickness of about 50–100 μm were prepared by casting from 3 wt% toluene solution (under ambient atmosphere) and subsequent thermal annealing at *T*=120 °C for 24 h. We note that this preparation route is similar to established fabrication methods of colloidal crystal structures, in which weak compressive forces are being harnessed to drive the close-packing of colloidal particles and the annealing of defects to yield highly ordered structures^18^. The resulting crystal-type particle brush assembly structure is a prerequisite for the application of scattering theory to interpret the mechanism of HG formation. However, in contrast to BG in regular hard-sphere colloidal crystals, the HG in particle brush films are robust with respect to structural disorder. To demonstrate this important feature, we also explored alternative preparation pathways such as rapid film casting or the mixing of distinct types of brush particles that result in only short-ranged ordered materials (see also Discussion below).

To assess the microstructure of particle brush assemblies, reference films of ∼1 μm thickness (corresponding to stacks of three or four particle brushes) were prepared following analogous procedures and analysed using TEM after film lift-off. Figure 2a depicts a representative bright-field TEM of a particle brush (*N*=1,000) multilayer revealing uniform fcc-type packing of spheres, identified by the characteristic sixfold contrast pattern of the <111> projection (Fig. 2b). The result suggests that the driving force for order formation in particle brush systems—at least for the materials and process conditions applied in the present work—resemble those of hard sphere systems for which fcc packing is typically observed^7^,^19^. Analysis of the particle surface-to-surface distance in particle monolayer structures (see inset of Fig. 2a for *N*=400) reveals the dependence of brush height on the degree of polymerization of surface-tethered chains *h*∼*N*^0.8±0.1^ thus confirming the pronounced stretching of surface-grafted chains^20^.

### Recording hypersonic phononic dispersion relations

The experimental Brillouin light scattering (BLS) spectra were recorded for both in-plane and out-of-plane phonon propagation through the selection of the wave vector direction (*q*_*||*_ or *q*_⊥_), as indicated by the two scattering configurations in Fig. 3a,b. In addition, polarized and depolarized BLS spectra were acquired with longitudinal (vv) and transverse (vh) polarizations (see Methods for details) to reveal the nature of the phonon propagation. In general, for homogeneous media, the transverse phonon is directly observed in the *I*_vh_(*ω*) spectrum, whereas the longitudinal phonon is observed in the isotropic *I*_iso_(*ω*)*=I*_vv_(*ω*)*-xI*_vh_(*ω*); for fluids^21^ *x*∼4/3. For the known cases, for example, hard sphere colloidal crystals, *I*_iso_(*ω*)*=I*_vv_(*ω*) because of the very weak intensity of the *I*_vh_(*ω*) spectrum^7^. The situation is very different in the present particle brush assemblies. Polarized (vv) and depolarized (vh) BLS spectra are shown in the insets of Fig. 3c at one representative *q*_*||*_ value near the observed BG for three particle brush systems. The *I*_vh_(*ω*) contribution to *I*_vv_(*ω*) is unexpectedly significant and the two spectra have qualitatively different shape. Although the former displays a single but complex peak structure with broad low-frequency wing, *I*_vv_(*ω*) has a double peak structure that becomes increasingly pronounced with increasing brush thickness as evidenced by the spectra of DP600 (Supplementary Fig. 1).

To account for the features revealed by the experimental spectra, we first consider the contribution of *I*_iso_(*ω*) that is obtained from *I*_vv_(*ω*) and *I*_vh_(*ω*) by adjusting the value of *x* between 0.7 and 4/3. This range of values was theoretically derived for fluids and was found to apply for the present spectra at long phonon wavelengths (low *q*_*||*_'s). The analysis unequivocally reveals that the unusual asymmetric shape of *I*_vv_(*ω*) at low frequencies is due to the *I*_vh_(*ω*) contribution. Its subtraction unravels the longitudinal phonon polarization character of the *I*_iso_(*ω*) spectra, which are well represented by two Lorentzian lines (red solid lines in inset of Fig. 3c). At low *q*_*||*_, a single Lorentzian fit is sufficient to represent the effective medium acoustic phonon (Supplementary Fig. 2). The dispersion plots constructed on the basis of the phonon frequency peaks are shown in Fig. 3c for three distinct particle brush systems. For the two propagation directions, *q*_*||*_ and *q*_⊥_, the dispersion relation is consistent with isotropic mechanical characteristics of film samples. Note that peak positions of *I*_vh_(*ω*) (open symbols in Fig. 3c) are both *q*_*||*_ and *q*_⊥_ independent, hence identifying the corresponding modes as localized modes in real space. Owing to the asymmetry of the *I*_vh_(*ω*) at the low-frequency side, the open symbols in the dispersion relation of Fig. 3c refer to the peak position of the *I*_vh_(*ω*).

**Figure 1:**
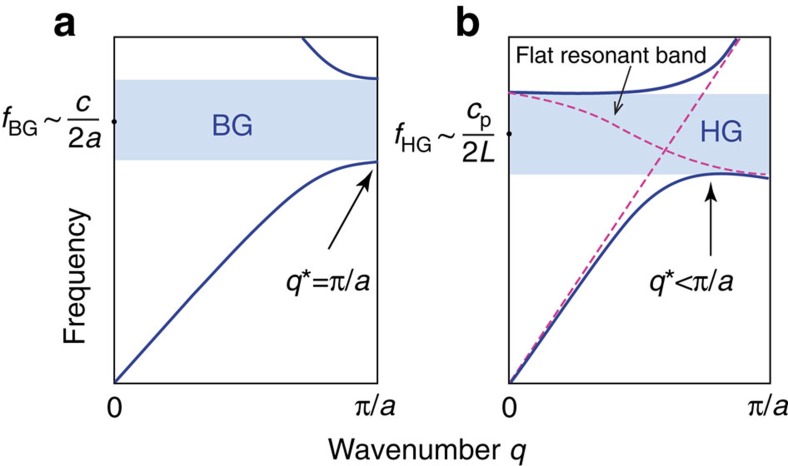
Schematic representation of Bragg and hybridization gaps. (**a**) Structure-directed, ‘Bragg' (BG) gap occurring at a frequency *f*∼*q*_BZ_ *c*/2*π* at the edge of the Brillouin zone (BZ), *q*_BZ_=*π*/*a*, where *c* is the sound velocity in the composite medium and *a* the lattice constant. (**b**) Hybridization (HG) gap is originating from an anti-crossing opening up at q*<*q*_BZ_ and involving a local resonant mode that occurs at a frequency *f*_HG_ related to the particle resonance characteristics (see the text).

The experimental phononic band diagrams *f*(*q*) in Fig. 3c display three pertinent features. (i) In the low *q*_*||*_ range the dispersion is linear (red solid lines); its slope determines the effective medium sound velocity *c*_eff_ that decreases with increasing PS fraction, as shown in Table 1. (ii) The spectra reveal a single phononic band gap at q*, with gap width Δ*f*_g_ that narrows with PS fraction. (iii) A new flat band (*q*-independent frequency, open circles) is present with almost exclusive transverse polarization. The frequency of the flat band (*f*_flat_) decreases with increasing particle size and an anti-crossing with the acoustic branch near the band gap is absent, as evidenced by its evolution in the four systems (see Fig. 3c and Supplementary Fig. 1 for details). The latter implies a different symmetry for each of the two bands (as confirmed by the vh and vv analysis), thus preventing a HG at this band-crossing region, unlike the behaviour shown in Fig. 1b. Furthermore, near the q* region there are three modes, in contrast to the experimental band structure of conventional colloidal crystals that display only the lower and upper gap edge frequencies^7^,^8^,^9^.

### Structure determination and degree of order

To clarify the origin of band gap formation in particle brush films (HG or BG), it is instructive to correlate the gap formation with the ‘degree of order' in these systems. The latter was estimated by evaluating the structural uniformity of particle brush monolayers—a summary of the ‘degree of order' (measured in terms of the width of the distribution of normalized Voronoi cell areas^20^) for all particle brush systems is shown in Supplementary Figs 3 and 4. The analysis reveals that gap formation is robust against variations of the degree of order in particle brush systems. To further test the effect of structural disorder, the dispersion relation was determined for mixed binary DP100/DP400 and DP400/DP600 (Supplementary Fig. 5) particle brush film structures and similarly for DP400/PS (*M*_w_=10 k) with 37.5 wt% PS particle brush/homopolymer blend systems, in which order is significantly reduced as compared with the respective uniform analogues (not shown here). In both cases, the phononic band gap is found in the proximity of the corresponding gap of pristine DP400, thus demonstrating its robustness with respect to disorder (Supplementary Fig. 5). The ‘robustness to disorder' is indirect evidence for the HG origin of the gap and constitutes a major advantage with respect to fabrication of PnC materials by self-assembly methods. An insight into the origin of HG formation is provided by the analysis of the band diagram of the underlying (idealized) crystal structure.

As revealed by TEM analysis, the colloidal films correspond to stacks of (111) layers of particles. Their periodic arrangement on a fcc lattice restricts the study of the dispersion relation *f*(*q*) only in the first Brillouin zone (BZ), which in this case is a truncated octahedron (Fig. 4a). Its centre, Γ, corresponds to wave vectors **q**=0 and the high-symmetry direction [111] is pointed from Γ to the zone hexagonal-face centres L, denoted equivalently as ΓL. For the scattering geometries of Fig. 3a, all possible experimental **q** vectors are confined in a plane perpendicular to ΓL, whose intersection with the BZ forms a hexagon (Fig. 4a). To reproduce theoretically, the experimentally obtained dispersion plots shown in Fig. 3c, one can select the direction of **q** along ΓM (Fig. 4a) corresponding to [112] with M denoting the edge centre of the hexagon.

### Modelling of phononic band diagrams

For the theoretical description of an infinite fcc crystal of SiO_2_-core particles embedded in a PS matrix, we use the layered-multiple-scattering formalism^22^. We first consider all materials to be homogeneous and isotropic with perfect boundary conditions (PBCs) applied across the SiO_2_–PS interface (see Fig. 4b for details). We use standard bulk values for PS except otherwise stated (mass density *ρ*=1,050 kg m^−3^, and longitudinal and transverse elastic velocities *c*_L_*=*2,350 m s^−1^ and *c*_T_=1,210 m s^−1^, respectively) and for SiO_2_ values fixed from previous study^23^ (*ρ*=1,850 kg m^−3^, *c*_L_=4,910 m s^−1^ and *c*_T_=3,090 m s^−1^). In Fig. 4c, we show the band structure diagram for the DP400 crystal (*d*=176 nm) along the high-symmetry direction ΓL, which is similar to the one along the low-symmetry direction ΓM. Because of its high symmetry, the choice of ΓL direction offers, apart from its numerical advantages (rapid calculation and convergence), a more straightforward interpretation of the symmetry^24^ and thus the polarization of the several eigenmodes: non-degenerate (Λ_1_ or Λ_2_) and double degenerate (Λ_3_), as explained in the Methods (Symmetry Considerations for Band Structure Analysis).

**Figure 2:**
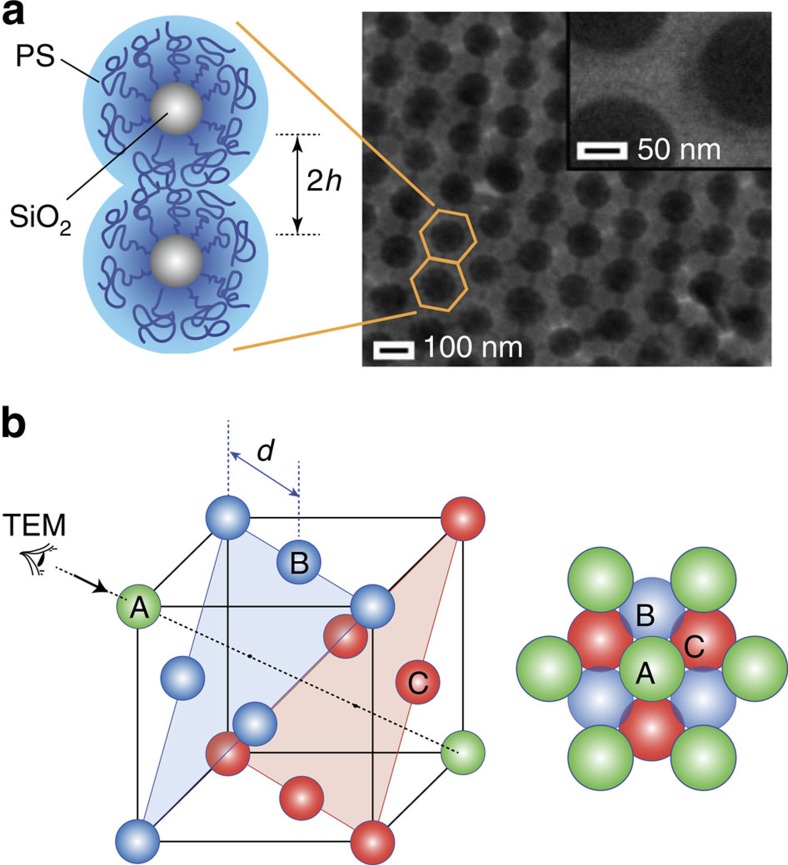
Structure of gradient-interface particle brush colloidal crystal. (**a**) Bright-field transmission electron microscopy image of particle brush film revealing projection of a four particle-stack. In the micrograph, the sixfold intersection of contrast regions is indicative of fcc packing. The inset shows a magnified image of individual brush particles enlightening the ‘inner shell' of extended polymer segments (faint ring in the image). The scheme illustrates the conformational transition and interdigitation of tethered polymer chains on silica spheres. (**b**) Schematic of fcc packing structure of brush particles with interparticle distance *d* along with an illustration of the projection image that is expected for fcc structure along [111] direction (black arrow in left panel).

The calculated PBC-based band diagram fails to describe both the flat experimental band at about 6 GHz and the higher acoustic branch, although the effective medium slope of the acoustic branch at the long wavelength limit is well reproduced. A small HG for longitudinal modes at about 8 GHz is observed in the vicinity of the BG frequency *f*_BG_, away from the experimental one (spanning from 6 to 7.5 GHz). We note that an attempt to mimic the radial evolution of the chains near the silica surface (Fig. 2a), through simple or multiple PS concentric shells with progressively varying elastic parameters, fails as well to describe the experimental behaviour.

To reduce the disparity between the calculated band structure and the experimental results, we introduce IBCs to account for anisotropic behaviour close to the SiO_2_–PS interface (Fig. 4b). This type of conditions has been recently applied to correctly reproduce the eigenmode frequencies of SiO_2_–PS particle brush powders in the air^23^. Although the use of the same stiffness values improves considerably the agreement between theory and experiment, a new readjustment of the PS elastic velocities (about 9% higher than those of bulk PS) and of tangential stiffness value, *k*_T_ (=0.0405 GPa nm^−1^), is needed to capture the experimental dispersion plot, as shown in Fig. 4d. The effective medium slope is mainly governed by the former, whereas the latter tunes the position of the flat (inactive) band. Clearly, nondegenerate bands are BLS active, whereas double degenerate ones are not easily discernible in the BLS spectra. The computed band structure along ΓM (Fig. 4e) reveals a better agreement between theory and experiment. In this case, because of the lower symmetry along [112], as compared with the high-symmetry [111], all bands are nondegenerate and BLS active to a greater or lesser degree depending on their hybrid polarization (for details, see the Methods section (Symmetry Considerations for Band Structure Analysis)).

To elucidate the origin of the flat band, density-of-states (DOS) calculations were performed first for an individual SiO_2_ particle (Fig. 5a) and subsequently for an infinite (111) array of particles (Fig. 5b) embedded within PS matrix. The IBC model reveals for the case of a single SiO_2_ particle the existence of a set of triple (=2*l*+1, due to spherical symmetry) degenerate dipole (*l*=1) modes corresponding to spheroidal (*f*=4.82 GHz) and torsional (*f*=5.20 GHz) polarization. The former exhibits a broad resonance peak implying a short lifetime and strong leakage outside the sphere, whereas the latter corresponds to rotational modes with the maximum intensity field occurring at the equator-level (inset of Fig. 5a) and well confined within the sphere (their narrow resonance peak implies a long lifetime). For an array of spheres in a (111) plane with interparticle distance *d*=201 nm (that is the DP600 case), the lower symmetry of the system with respect to the single spherical particle leads to a level splitting of each triple state into a double degenerate and a single nondegenerate (Fig. 5d), as confirmed by the corresponding DOS calculation (Fig. 5b). Indeed, we observe three relatively narrow resonance modes, two of them double degenerate (*f*=5.32 and 6.78 GHz) and one nondegenerate (*f*=6.24 GHz), as well as one quasi-bound extremely narrow peak at *f*=5.24 GHz. We note that this mode is strictly bound (a delta function in DOS spectra) at normal incidence, and becomes active (a narrow Lorentzian-shaped peak in DOS) at a slightly-off normal incidence.

At resonance frequency, the corresponding displacement field intensity plot within the unit cell and at a plane at the centres of the spheres (*z*=0) points out a strong localization inside the particle and close to its surface (inset of Fig. 5b), decaying away from this plane. The almost exclusive rotational character originates from the torsional polarization of the single sphere mode. Its strongly localized nature leads to a very weak interaction when (111) planes are combined to form the crystal. Consequently, it results in a very flat resonance band of Λ_2_ symmetry, as can be seen in Fig. 5c, with a frequency approximately equal to the individual particle's torsional mode (Fig. 5d). On the contrary, the strong leakage of the rest of the (111) plane modes leads to much broader resonance bands of Λ_1_/Λ_3_ symmetry, for the fcc crystal along ΓL direction. They interact with the same-symmetry propagating bands that describe the effective medium assembly; this avoided crossing gives rise to the corresponding HG, which is shown in Fig. 5c. We note that the flat band indicates also the low-frequency limit of the first HG for transverse modes. The observation of the highly localized flat band for the first time at submicrometre scale could find application as a flag for transverse wave filtering. On the other hand, these highly localized rotational modes could be of importance to future applications where energy harvesting inside the spheres is necessary. The theoretical analysis, apart from its obvious importance for designing tunable robust hypersonic HGs, unravels these features of potential interest.

## Discussion

The characteristics of elastic wave propagation in particle brush assembly structures are distinctively different from those of known systems with low-density mismatch components (silica in polymer is a typical candidate) where HGs occur in the vicinity of the first BG for longitudinal modes (Fig. 4c). These densely polymer-tethered particle assemblies exhibit—thanks to their local anisotropy at the surface that is modelled here by IBC—HGs that can be deliberately tuned by modification of the brush height for a given silica-core size towards lower frequencies (Supplementary Fig. 6). This is remarkable since up to now this effect has only been known to exist in high-density mismatch components (like metal-in-polymer composites). In the present theoretical framework, the experimental dispersion relations can only be captured by adjustment of sound velocities of PS and the tangential stiffness across the particle/polymer interface. To our knowledge, the control of the HG characteristics (frequency position and width) by introducing ‘interface elastic anisotropy' has never been demonstrated before and its features strongly suggest a ‘new class' of HG-phononic materials. The first evidence (shown in Fig. 6a) are the frequencies of the gap region and the decreasing of the gap-width of the HG with increasing degree of polymerization (*N*) of the grafted chains. This trend reflects a ‘dilution effect' because of the decrease of the SiO_2_ core volume fraction.

Second, the assumed value of the longitudinal sound velocity for PS *c*_PS_ (Table 1) equals the corresponding bulk PS value (*c*_L_) only for the longest PS graft (DP1000). For the shorter grafts, *c*_PS_ exceeds *c*_L_ and the difference increases to 20% for the shortest PS graft (DP100). This systematic trend with graft length (Table 1) is consistent with the increasingly (radially) stretched chain conformation that is expected for shorter brush lengths. Our results indicate that the pronounced stretching of surface-grafted chains leads to strain hardening, resulting from limited chain extensibility that yields the increase in the modulus (and hence *c*_PS_) normal to the interface. We note that this observation supports a prior report of ‘stiffening' in planar polymer brushes^25^. Furthermore, the assumed values of *c*_PS_ reproduce (within 1%) the effective medium velocity *c*_eff_ (6th and 7th columns in Table 1). As expected *c*_eff_ is less than the matrix sound velocity (*c*_PS_) for solid (SiO_2_) inclusions in solid matrices (PS), which support shear waves^26^. The same trend is theoretically predicted in the case of all-solid composites involving PBC^27^, although these models fail to describe quantitatively IBC-type systems.

**Figure 3:**
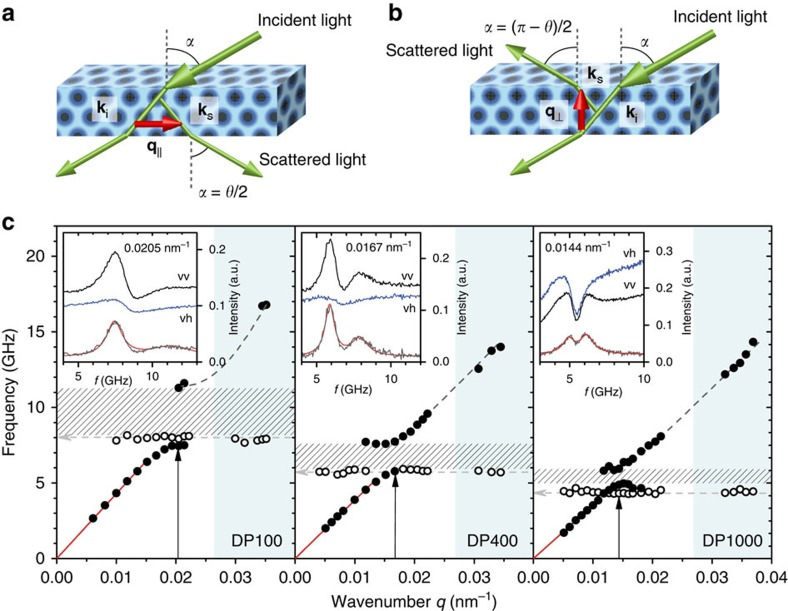
Recording the phononic band diagram. (**a**,**b**) Brillouin light scattering geometries probing phonon propagation along the wave vector **q=k**_s_–**k**_i_, with **k**_s_, **k**_i_ being, respectively, the incident laser and scattered light wave vector. The direction of **q** is selected either in-plane ((**a**) transmission geometry) or normal to the plane ((**b**) reflection geometry). In the transmission geometry, the magnitude *q* is tuned by varying the scattering angle *θ* and is independent of the refractive index of the medium. (**c**) Experimental dispersion relation (frequency versus wavenumber *q*) for DP100, DP400 and DP1000 samples, obtained from the corresponding deducted BLS spectra (insets) recorded at a given *q* (vertical arrows) and fitted as a sum of Lorentzian shapes (red lines). The deducted isotropic (grey) spectra is the difference between the intensities recorded in vv (black) and vh (blue) polarizations, *I*_vv_*-xI*_vh_, with *x* being a variable factor between 0.7 and 4/3. In each plot, a clear band gap region (patterned area) and a localized mode (open circles) are observed. The red lines in the low *q* regime represent the effective medium acoustic mode; the dashed grey lines in the high-frequency branch are guides to the eye, connecting the data acquired with **q** perpendicular to the substrate plane (blue shaded area) using the reflection geometry in **a**. The frequency of the flat mode is indicated by a grey dashed arrow, and wavenumber number of the gap opening is marked with a black solid arrow, with an error of ∼0.002 nm^−1^.

**Figure 4:**
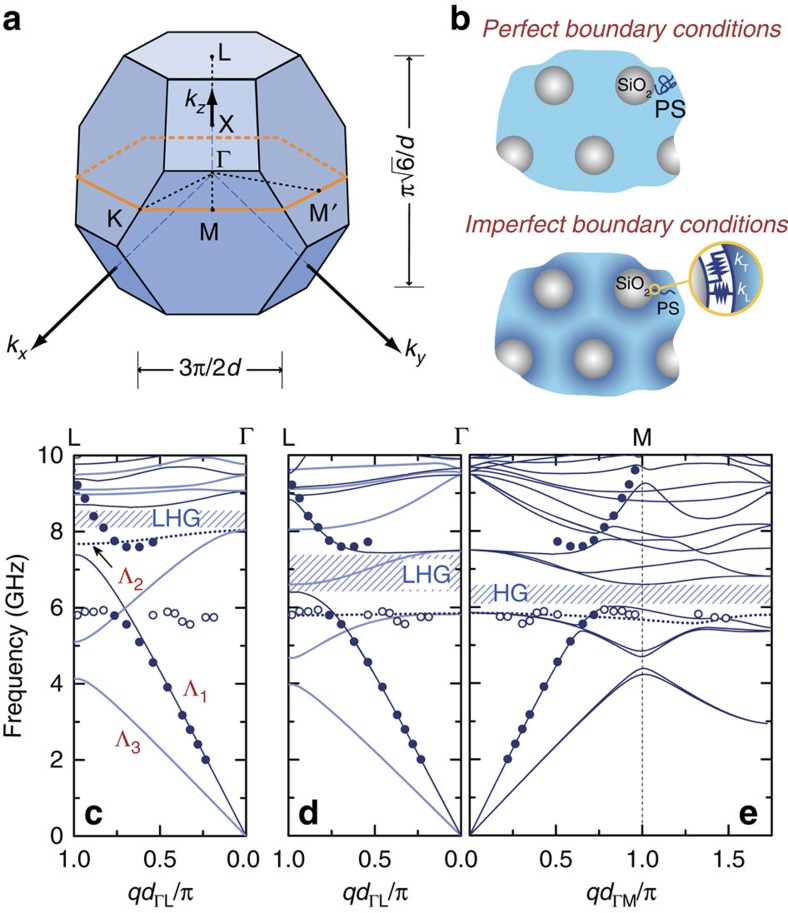
Theoretical versus experimental band diagrams. (**a**) Reciprocal fcc lattice showing a [112] plane (orange hexagon) and the ΓM direction probed in the experiment. (**b**) Schematic presentation of PBC and IBC models applied at the SiO_2_-PS interface. In the IBC model, the springs of respective stiffness *k*_L,T_ represent the discontinuity of the displacement field across this interface. The theoretical band diagram of the DP400 is shown for PBC along [111] with the bulk PS sound velocities in **c** and for IBC with PS velocities (*c*_L_=2,560 m s^−1^, *c*_T_=1,320 m s^−1^) about 9% higher than bulk PS along [111] (**d**) and [112] (**e**). Along ΓL dark/light solid and dotted blue lines denote non-degenerate (longitudinal), double degenerate (transverse) and deaf computed bands, respectively. In **e**, all bands are non-degenerate of mixed character; the flat band is highlighted (dotted line). Solid and open circles indicate the experimental points (see Fig. 3). Hatched regions denote hybridization gaps (LHG for longitudinal modes; HG for all modes).

**Figure 5:**
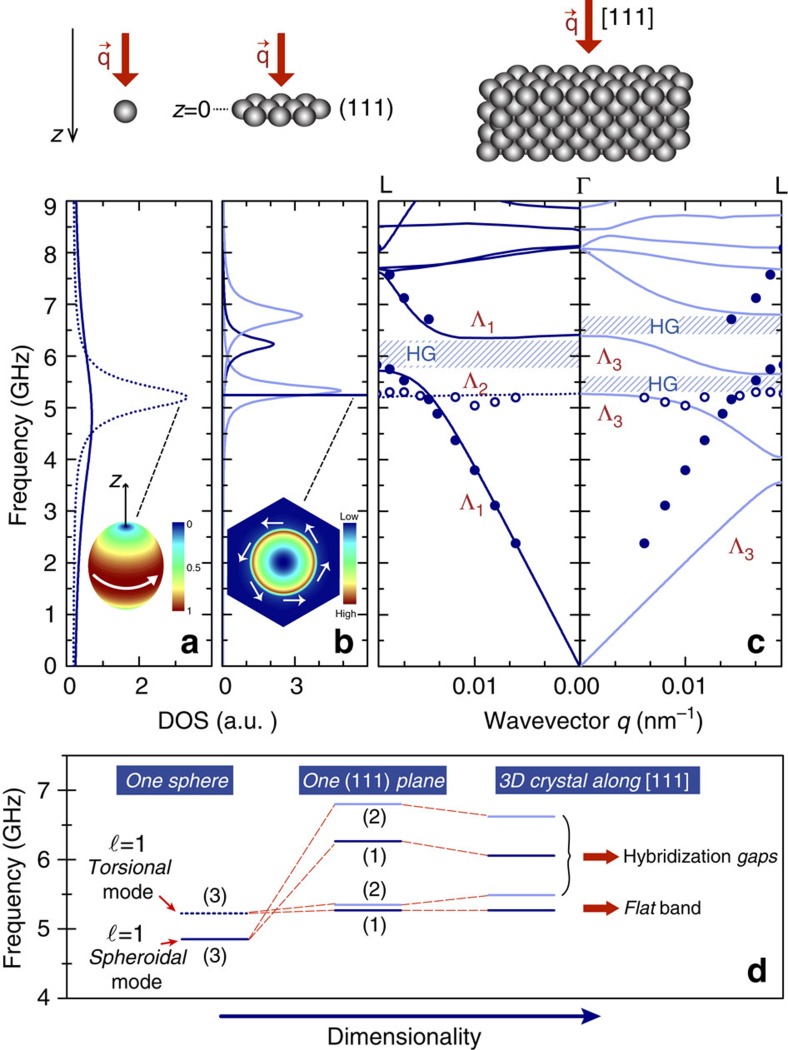
Physical origin of the dispersion characteristics and their evolutive formation with dimensionality. Calculated DOS for (**a**) one SiO_2_ particle in PS (solid/broken lines spheroidal/torsional *l*=1 modes) and (**b**) for one (111) plane of particles of the DP600 film at slightly off-normal incidence (dark/light blue lines: non-degenerate/double degenerate modes). The mode corresponding to the very sharp peak in DOS plot of (111) plane, is associated to the flat band of the crystal (see **c**). Its field intensity representation within the unit cell passing at the centre of the sphere (*z*=0), at *f*=5.24 GHz, is shown in the inset. White arrows represent the quasi-pure rotational character of the field everywhere in the unit cell. (**c**) The band structure of the corresponding crystal along [111], with non-degenerate (left) and double degenerate (right) calculated bands together with the experimental points (symbols). The notation used is that of Fig. 3. (**d**) Schematic representation of the evolution of these modes when passing from a single sphere to the whole crystal. Parenthesized numbers denote the number of states per level. In the case of three-dimensional (3D) crystal, the levels indicating the hybridized modes correspond to HG centres.

**Figure 6:**
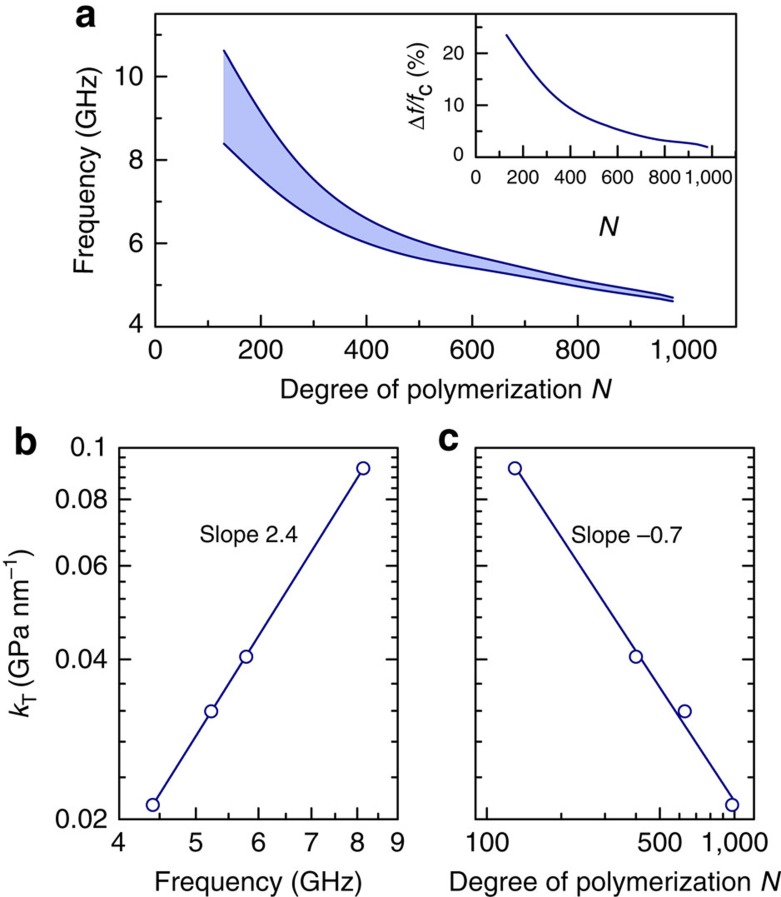
Controlling the core-brush assemblies behaviour. (**a**) Evolution of the frequency gap region (shaded area) for all modes with *N*, following the theoretical predictions (see the text) along ΓM. Inset: normalized gap width (*f*_c_ being the central frequency of the gap). Variation of the tangential stiffness values *k*_T_ with (**b**) the frequency of the flat band *f*_flat_ calculated in the middle of the band along ΓM (*qd*_ΓM_=0.5*π*), and with (**c**) the degree of polymerization *N* of the assemblies considered, showing a power-law behaviour in both cases.

Third, the quantity *k*_T_ captures the increase of the flat mode frequency (*f*_flat_) with decreasing degree of polymerization, that is, from DP100 to DP1000 (Fig. 3c and Supplementary Fig. 1). This trend is visualized in Fig. 6b,c. In view of the proposed mechanism (Fig. 5d), the coupling of the core eigenmode should be stronger for larger stiffness constant and hence the observed scaling in Fig. 6b should be system dependent. The dependence of *k*_T_ with *N* is depicted in Fig. 6c. Interestingly, the dependence of stiffness constant on the degree of polymerization of grafted chains is found to be described by the scaling relation *k*_T_∼*N*^−0.7^. Although the decrease of *k*_T_ with increasing *N* could be anticipated, the particular scaling is an unexpected finding. It reflects an almost reciprocal relationship to the brush height (*h*∼*N*^0.8±0.1^) and suggests a direct relation between the chain conformational anisotropy and the apparent interfacial stiffness. Although a more detailed understanding of the origin of the scaling relationship is currently lacking, it is important to point out its role as a ‘simple' design guideline for the synthesis of particle brush materials with engineered phononic hybridization gap.

In conclusion, we have demonstrated that the self-assembly of densely polymer-tethered colloidal particles gives rise to phononic materials with HGs that are robust to disorder. Theoretical analysis reveals that the origin of HG formation in particle brush assemblies is the hybridization of local resonant modes controlled by the anisotropic elastic coupling across the particle/brush interface. The analysis further reveals that the phononic properties can deliberately be tuned by variation of particle (and polymer) composition, particle size and the degree of polymerization of tethered chains. In contrast to established metamaterial systems, the new process does not require expensive microfabrication but can rather be accomplished by the dense tethering of polymeric chains to the surface of colloidal particles. The ability to synthesize suitable core-shell particles using economic polymerization methods that are already being used on industrial scale in conjunction with the formability of particle brush materials and the demonstrated robustness of HG, renders this new material approach an intriguing candidate for the viable fabrication of phononic hybrid materials promoting the development of a wide range of innovative phononic material technologies.

## Methods

### Particle brush synthesis

Silica particles (*R*_c_=57±6 nm) were obtained from Nissan Chemicals. Styrene (S) was obtained from Aldrich and purified by passing through an alumina-filled column. The synthesis of PS-grafted particle brush systems was performed using surface-initiated atom transfer radical polymerization as described previously^16^,^17^. The polymer grafting density *σ*=0.5–0.6 chains per nm^2^ was determined by elemental analysis as well as thermogravimetry. The molecular weight distribution of surface-grafted chains was determined by size exclusion chromatography after dissolution of the particle core in hydrofluoric acid. The graft characteristics of the particle systems, grafting density *σ*, degree of polymerization *N* (=130–980) of the surface tethered chains are listed in Table 1.

### Brillouin spectroscopy

Brillouin spectroscopy utilizes the scattering of a probe laser beam from thermally activated phonons along a specified direction defined by the scattering vector **q**. The magnitude of the scattering vector, defined as **q**=**k**_s_-**k**_i_, is independent from the refractive index in transmission geometry (*q*_*||*_=4*π*/*λ* sin[*θ*/2], *θ* is the scattering angle and *λ*=532 nm is the wavelength of the incident light, Fig. 3a), whereas in reflection geometry, *q*_*⊥*_(*n*) depends on the refractive index (Fig. 3b). The inelastic interaction between the incident photons and thermal phonons is evidenced in the frequency shift *f*(*q*) of the BLS spectrum at hypersonic (GHz) frequencies, resolved by a high-resolution tandem Fabry–Perot interferometer (JRS Instruments). Longitudinal (transverse) displacements have associated a vv (vh) polarization, selected by the input polarizer (v) and output analyser (v or h); v(h) denotes vertically (horizontally) polarized light relatively to the scattered plane.

### Theoretical calculations

Multiple-scattering theory is applied to describe the propagation of elastic waves in the colloidal assemblies and calculate the dispersion relation (band structure) for such periodic systems^22^. The essence of this method consists in the multipole expansion of the elastic field in each region containing an isotropic and homogeneous material. Discontinuities are treated through interfaces on which appropriate boundary conditions (BCs) are applied. For instance, in the case of an ensemble of spherical bodies embedded in a host, the elastic field is expanded into a basis of spherical waves characterized by the angular momentum *l*, the azimuthal number *m* and by the polarization P (longitudinal L, and transverse, M or N). For a single sphere, the elastic eigenmodes are organized in two independent (uncoupled) subgroups: the torsional modes (M polarized) and the spheroidal modes (L and N polarized). The application of the appropriate boundary conditions on the surface of the spherical particle leads to the scattered field by that particle, described by the corresponding transition **T**-matrix. When bringing together the ensemble of particles, one needs to consider the auto-consistent field derived by the multiple-scattering process of elastic waves between the particles, in order to describe accurately the scattering by the aggregate; key quantities are the propagation matrix **Ω** and the **T**-matrix of the individual particles. The difference in the DOS of the elastic field with respect to the infinite host matrix (PS), for one particle or an ensemble of such particles, is given by 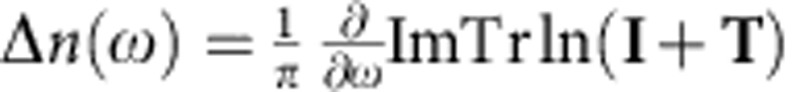 and 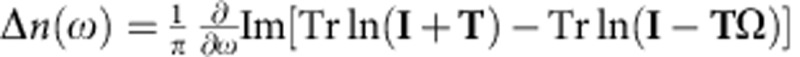, respectively^26^. The dispersion relation of the elastic modes of a crystal composed by these particles is also derived from these two key matrices, **T** and **I**−**TΩ**. Here, we apply two different types of boundary conditions on the interface between SiO_2_ cores and PS host: (i) PBCs assuming continuity of all radial and tangential components of the displacement field and surface traction, and (ii) IBCs introducing discontinuity of the displacement field components across the interface (surface traction remains continuous) through a stiffness coefficient *k*. Note that *k* is different for the displacement components normal (*k*_L_) and tangential (*k*_T_) to the spherical surface. This model was originally developed for cylindrical inclusions^28^ and was recently adapted for spherical particles^29^. It has been successfully compared with experimental data for individual core-brush particles in the air^23^.

### Symmetry considerations for band structure analysis

Following a group theory analysis^24^, only three different-symmetry groups of bands are observed: double degenerate active (Λ_3_) and nondegenerate active (Λ_1_) bands whose modes are excited by a transverse and longitudinal wave incident normally on a finite (111) slab of the crystal (that is, along ΓL), and nondegenerate inactive (deaf, Λ_2_) bands whose modes cannot be excited by any type of elastic waves propagating along ΓL. The latter become active when longitudinal waves are incident on the structure at a slightly off-normal incidence to the finite (111) slab of the crystal (that is, along a direction slightly different from ΓL). In a real experiment, all nondegenerate bands will become active (although not to the same degree), as experimentally small angular deviations from a specific direction of propagation—here ΓL—always exist. Therefore, one expects that these non-degenerate bands are BLS active in our case. Double degenerate bands are associated to modes coupled to transverse elastic waves incident on the slab. One expects that these double degenerate bands are BLS inactive along ΓL direction. For directions of lower symmetry, the above scheme is no longer valid. For instance, along ΓM (which is different from but close to ΓL) all bands become non-degenerate, but their modes ‘remember' the polarization character along ΓL: they are now hybrid but with a large percentage remaining in the polarization from the corresponding high-symmetry branch.

## Additional information

**How to cite this article:** Alonso-Redondo, E. *et al*. A new class of tunable hypersonic phononic crystals based on polymer-tethered colloids. *Nat. Commun.* 6:8309 doi: 10.1038/ncomms9309 (2015).

## Supplementary Material

Supplementary Information Supplementary Figures 1-6

## Figures and Tables

**Table 1:** Samples characteristics and longitudinal sound velocities in the assemblies.

**Sample**	***σ*** **(nm**^**−2**^**)**	***N***	***d*** **(nm)**	***c***_**PS**_ **(m s^−1^)**	***c***_**eff-th**_ **(m s^−1^)**	***c***_**eff-exp**_ **(m s^−1^)**
DP100	0.61	130	142	2,820	2,688	2,710
DP400	0.61	400	176	2,562	2,482	2,480
DP600	0.56	630	201	2,526	2,469	2,420
DP1000	0.48	980	214	2,350	2,309	2,360

*σ*, grafting density; *N*, degree of polymerization; *d*, distance core to core; *c*_PS_, polystyrene sound velocity; *c*_eff,th_, computed effective sound velocity; *c*_eff,exp_, measured effective sound velocity.
