# Supplementary material for: A new class of tunable hypersonic phononic crystals based on polymer-tethered colloids
Source: Nat Commun. 2015 Sep 22;6:8309. doi: 10.1038/ncomms9309 (PMC4595630; doi:10.1038/ncomms9309)
Supplement: Supplementary Information — Supplementary Figures 1-6 [file ncomms9309-s1.pdf]

Supplementary Figures

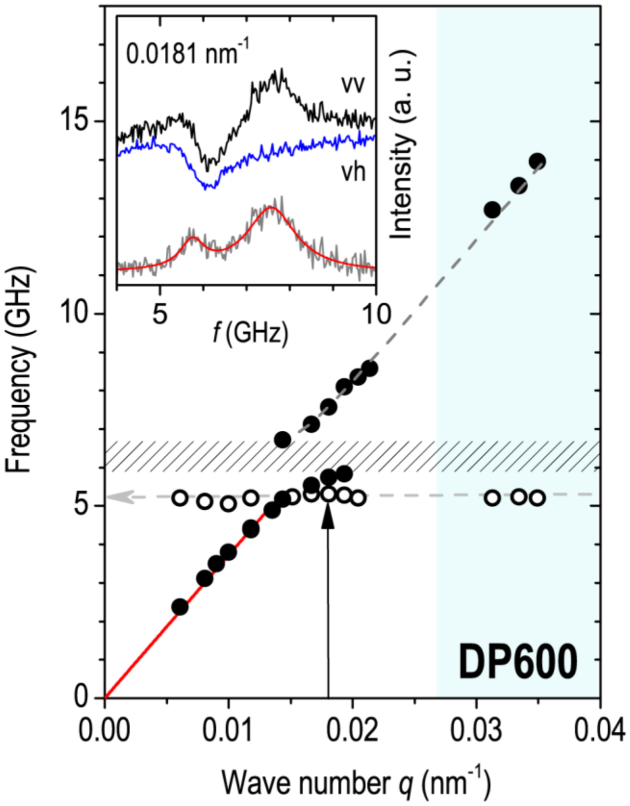

Supplementary Figure 1. Phononic band diagram of DP600 system. Experimental dispersion relation for DP600 sample, obtained from the polarized (vv) and depolarized (vh) BLS spectra recorded at several wave numbers q in parallel and normal (blue shaded area) to the film. Inset: BLS spectra at a single q, pointed by a vertical arrow in the dispersion. The isotropic spectra (grey) is the difference between the intensities recorded in vv (black) and vh (blue) polarizations, and is represented by the sum of two Lorentzian shapes (red solid line). Solid and open symbols in the dispersion plot are obtained from the isotropic and vh spectra respectively. The red straight line in the low q regime is the linear fitting of the first points, and its slope provides the effective medium sound velocity. The dashed grey lines in the branch above the gap region and around the flat mode are guides to the eye.

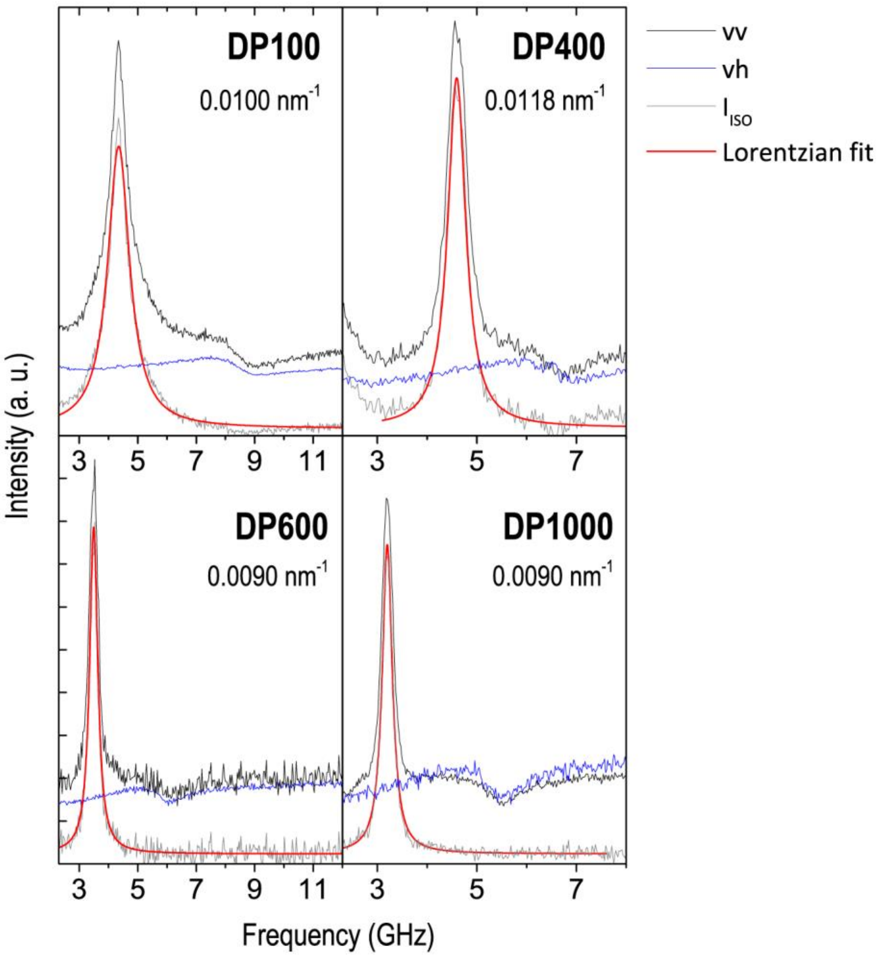

Supplementary Figure 2. Experimental spectra in the low wave number regime. Experimental BLS spectra for the four systems DPN (N = 100, 400, 600, 1000), acquired at low wavenumbers q (indicated in every case) parallel to the film plane. The spectra were acquired in vv (black) and vh (blue) scattering polarizations, and the isotropic spectra (grey) are represented by single Lorentzian shapes (red solid lines).

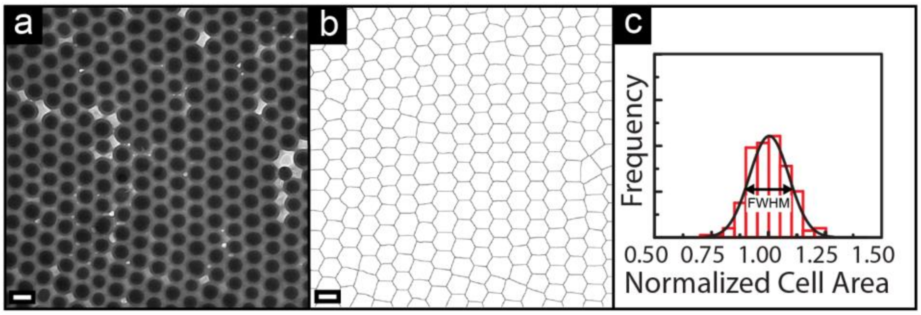

Supplementary Figure 3. Degree of order method. Illustration of the process used to determine the 'degree of order' in particle brush monolayers. In a first step a particle brush monolayer is cast on poly (acrylic acid) (PAA) substrate. The film is lifted-off by immersion of substrate in water after thermal annealing for 48 h at 140 ºC and (**a**) imaged using transmission electron microscopy. (**b**) Micrographs of particle brush monolayers are processed by Voronoi tessellation. (**c**) The 'degree of order' is determined as the full-width-at-half-maximum (FWHM) of the distribution of normalized Voronoi cell areas. The continuous black line represents the fit of the normal distribution to the experimental data. Scale bars correspond to 200 nm.

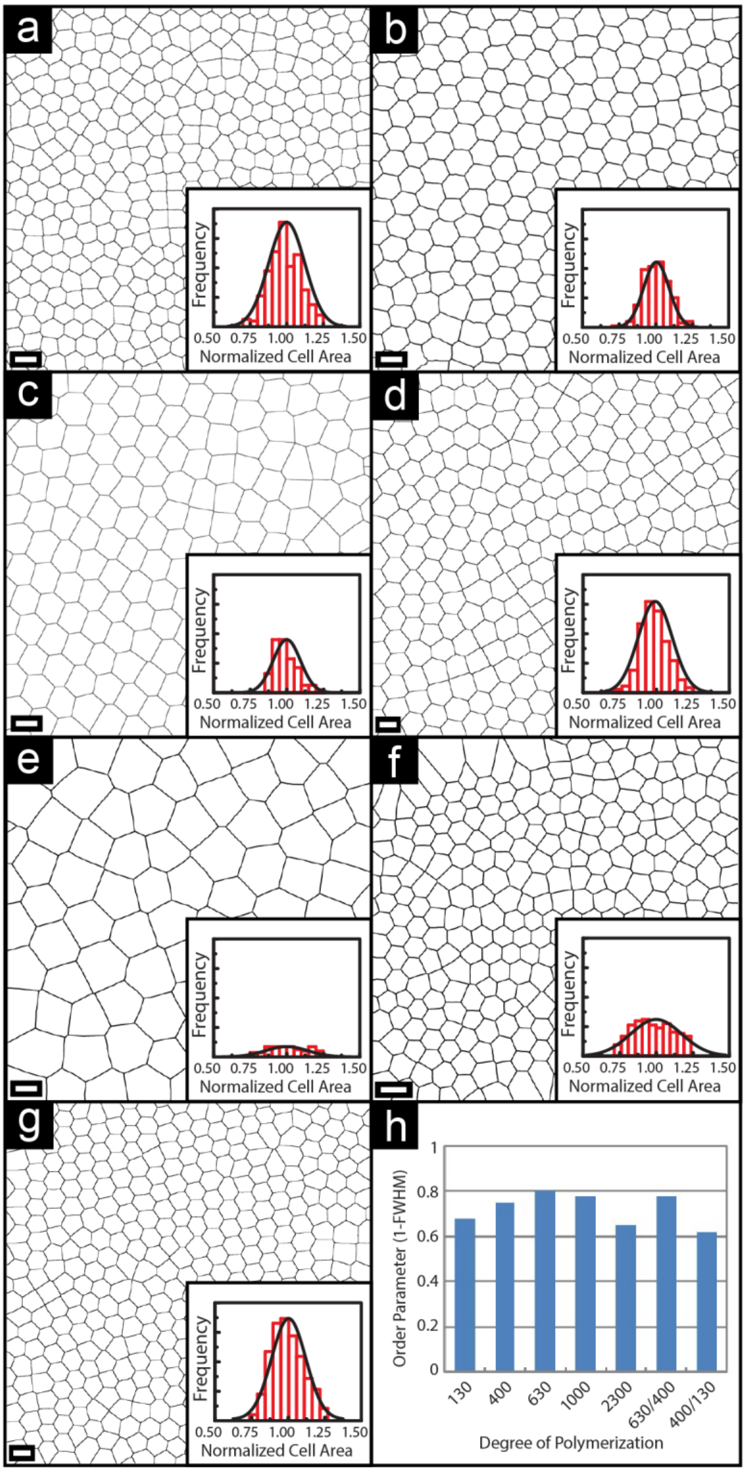

Supplementary Figure 4. Degree of order in mixed binary particle brush films and particle brush/homopolymer blend systems. Comparison of 'degree of order' in uniform and mixed particle brush monolayer film structures. Panels show Voronoi tessellations along with corresponding distribution of normalized Voronoi cell areas (with solid lines representing fits of the normal distribution to the experimental data). (**a**) DP100; (**b**): DP400; (**c**) DP600; (**d**) DP1000; (**e**) DP2300; (**f**) DP400/DP100; (**g**) DP400/DP600; (**h**)

Comparison of ‘degree of order’ parameter (1-FWHM) for different particle brush systems. Degree of order is found to peak with the degree of polymerization of surface-tethered chains then decrease around the critical degree of polymerization for the stretched to relaxed conformation transition. The degree of order in mixed particle brush systems decreases comparatively to the respective homogeneous component systems. All scale bars correspond to 200 nm.

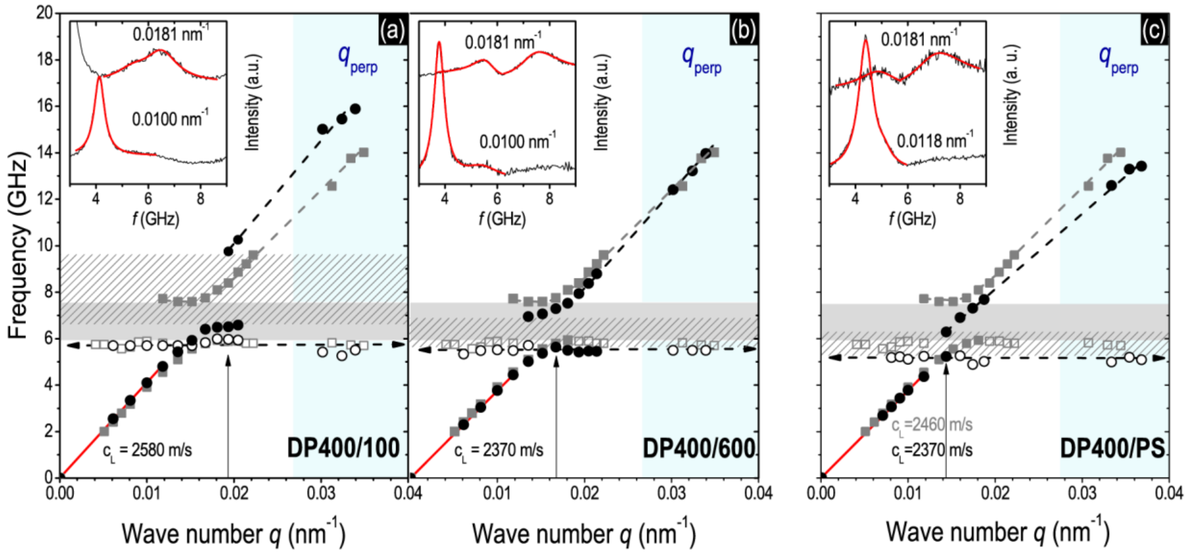

Supplementary Figure 5. Phononic band diagrams for binary systems. Experimental dispersion relation for (**a**) binary mixture of DP400 and DP100 particles with equal composition and (**b***)* DP400 and DP600 particle mixture obtained from the polarized (vv) and depolarized (vh) BLS spectra. Black solid circles are phonon frequencies from polarized spectra in-plane and out-of-plane (blue shaded region), and the open circles correspond to the flat mode frequency (taken from vh). The dotted horizontal black line shows the average flat band frequency, the black arrow points to q^* for the system, the red line at low q is a linear representation that gives the effective medium sound velocity (as shown on the graph), the hatched rectangles represent the band gap region, and the dotted lines above the gap are to guide the eye. (**c**) The phononic band diagram of DP400 polymer tethered colloids mixed with 37.5% by mass homopolymer PS is shown by black solid circles and open circles (for the flat mode frequency). The grey solid and open squares are the dispersion relation for just the DP400 colloids (used as reference in all dispersion graphs). The grey horizontal rectangle is the band gap of DP400 (used in all graphs as well) and the hatched rectangle is the band gap of the DP400/PS blend. The upper left insets in each dispersion show spectra (black line) at two different wave vectors, represented by Lorentzian shapes (red line).

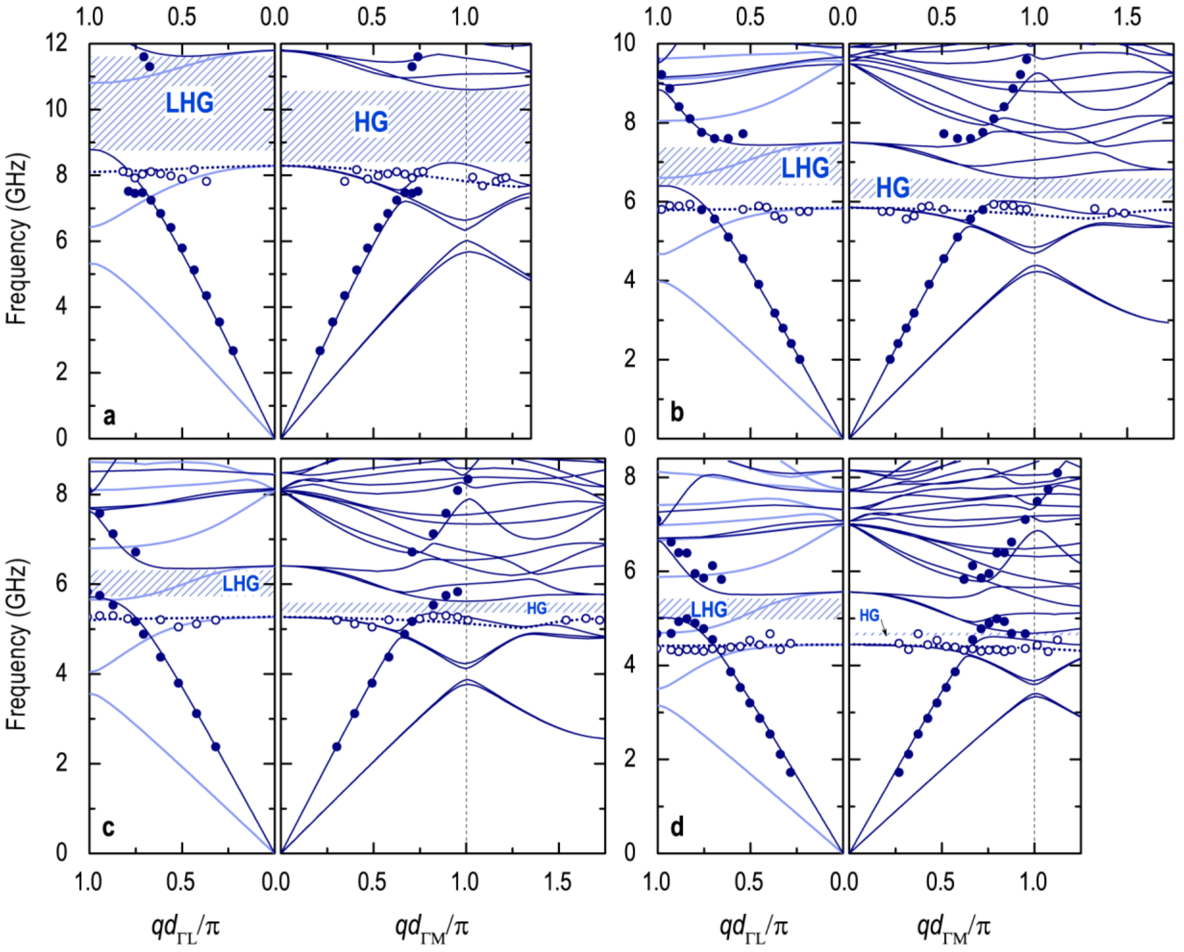

Supplementary Figure 6. Computed phononic band diagrams. Computed band diagrams along ΓL and ΓM (left and right panels respectively of each subplot) for a fcc crystal of silica particles in PS matrix with IBC applied at the silica core-PS interface. In the calculations PS elastic velocities c_L and c_T as well as tangential stiffness coefficient k_T are adjusted to fit the corresponding experimental data. Four cases are considered: (**a**) DP100 (c_L = 2820 m s^{-1}, c_T = 1450 m s^{-1} –about 20% higher than bulk PS– and k_T = 0.0916 GPa nm^{-1}), (**b**) DP400 (c_L = 2560 m s^{-1}, c_T = 1320 m s^{-1} –about 9% higher than bulk PS– and k_T = 0.0405 GPa nm^{-1}), (**c**) DP600 (c_L = 2530 m s^{-1}, c_T = 1300 m s^{-1} –about 7.5% higher than bulk PS– and k_T = 0.0319 GPa nm^{-1}), and (**d**) DP1000 (c_L = 2350 m s^{-1}, c_T = 1210 m s^{-1} –equal to bulk velocity values– and k_T = 0.0213 GPa nm^{-1}). Dark and light solid lines denote non-degenerate and double-degenerate bands respectively; with dotted lines we denote the quasi-flat (highly localized) bands originating from dipole torsional modes (see main text). Solid and open circles indicate the experimental points, following the same nomenclature as in Supplementary Fig. 5. Hatched regions denote hybridization gaps (LHG for longitudinal modes; HG for all modes).
